# Corncob Cellulose Scaffolds: A New Sustainable Temporary Implant for Cartilage Replacement

**DOI:** 10.3390/jfb13020063

**Published:** 2022-05-23

**Authors:** Rachel Cordeiro, Marta Henriques, João C. Silva, Filipe Antunes, Nuno Alves, Carla Moura

**Affiliations:** 1 Centre for Rapid and Sustainable Product Development, Polytechnic of Leiria, 2430-028 Marinha Grande, Portugal; rachel.s.cordeiro@ipleiria.pt (R.C.); joao.f.da.silva@ist.utl.pt (J.C.S.); nuno.alves@ipleiria.pt (N.A.); 2 Polytechnic Institute of Coimbra, Coimbra Agriculture School, 3045-601 Coimbra, Portugal; mhenriques@esac.pt; 3 Research Centre for Natural Resources, Environment and Society (CERNAS), Polytechnic Institute of Coimbra, 3045-601 Coimbra, Portugal; 4 IBB—Institute for Bioengineering and Biosciences and Department of Bioengineering, Instituto Superior Técnico, Universidade de Lisboa, Av. Rovisco Pais, 1049-001 Lisboa, Portugal; 5 Associate Laboratory i4HB—Institute for Health and Bioeconomy, Instituto Superior Técnico, Universidade de Lisboa, Av. Rovisco Pais, 1049-001 Lisboa, Portugal; 6 Coimbra Chemistry Centre, Department of Chemistry, University of Coimbra, 3004-535 Coimbra, Portugal; filipe.antunes@ci.uc.pt; 7 Science 351, Instituto Pedro Nunes, Ed C, 3030-199 Coimbra, Portugal

**Keywords:** cartilage repair, corncob cellulose, scaffold, tissue engineering, sustainability

## Abstract

Tissue engineering using scaffolds is a promising strategy to repair damaged articular cartilage, whose self-repair is inefficient. Cellulose properties have been recognized for their application in the biomedical field. The aim of this study was to fabricate and characterize novel scaffolds based on poly(ɛ-caprolactone) (PCL) and sustainable cellulose. Thus, the performance of corncob-derived cellulose (CC) in scaffolds as an alternative to wood cellulose (WC) was also investigated to reduce the environmental footprint. Two concentrations of CC in scaffolds were tested, 1% and 2% (*w/w*), and commercial WC using the same concentrations, as a control. Morphologically, all the developed scaffolds presented pore sizes of ~300 µm, 10 layers, a circular shape and well-dispersed cellulose. Thus, all of these characteristics and properties provide the manufactured scaffolds suitable for use in cartilage-replacement strategies. The use of 2% CC results in higher porosity (54.24%), which promotes cell infiltration/migration and nutrient exchange, and has similar mechanical properties to WC. As for the effects of enzymatic degradation of the scaffolds, no significant changes (*p* > 0.05) were observed in resistance over time. However, the obtained compressive modulus of the scaffold with 2% CC was similar to that of WC. Overall, our results suggest that the integration of 2% corncob cellulose in PCL scaffolds could be a novel way to replace wood-cellulose-containing scaffolds, highlighting its potential for cartilage-replacement strategies.

## 1. Introduction

Osteoarthritis and rheumatoid arthritis are the most common musculoskeletal disorders affecting articular cartilage [1,2]. Data from 2017 indicated that, worldwide, the prevalence of osteoarthritis was around 303 million people, and of these, ca. 263 million were knee osteoarthritis [3]. The self-repair of cartilage is ineffective, as this tissue is avascular and the chondrocytes, if disordered, produce fibrocartilaginous tissue, which has lower mechanical properties and is predisposed to the progression of arthritis [4]. Currently, the available treatments for cartilage repair include techniques such as palliative therapies, microfracture, autologous chondrocyte implantation, osteochondral autograft transfer, osteochondral allograft transplantation, or scaffold-based techniques [5,6,7]. This latter technique relies on tissue engineering (TE), which provides a promising strategy to replace damaged cartilage with a long-lasting temporary implant [8,9,10]. The structure of this implant must offer mechanical support and ensure that it degrades while new tissue is formed [11]. Scaffolds must be three-dimensional (3D) with an interconnected pore structure and tunable size, mechanical properties, and degradation rates to meet the specific requirements of the target tissue. Moreover, a scaffold has to be biocompatible and promote cell adhesion and proliferation [12,13]. Different techniques for scaffold production can be used such as freeze drying, electrospinning, 3D printing or stereolithography. With these techniques, electrospinning meshes, rigid structures, sponges and even hydrogels/aerogels can be obtained. However, meshes, hydrogels/aerogels and sponges are not very effective due to their limited compressive strength [14,15].

Cartilage has unique characteristics, constituting a major challenge in TE approaches. One of these fundamental characteristics is mechanical resistance, which is believed to be overcome through a good support structure. Scaffolds manufactured by 3D printing with different biomaterials have the necessary support characteristics to withstand the forces exerted after its implantation, mimicking the mechanical resistance of the native tissue while allowing cell adhesion, proliferation and differentiation [16,17,18].

Cellulose is recognized as a natural polysaccharide that promotes cell adhesion. Its major contribution to the nanostructure of tissue-engineering scaffolds and impact on their macroscale mechanical properties has also been highlighted [19,20]. In addition, it has good biocompatibility with the human body, low toxicity, high surface area, resistance and rheological properties while being a renewable material. Thus, in the recent years, cellulose has experienced an increasing interest in the tissue-engineering and regenerative-medicine fields, with applications in skin [21], bone [22], nerves [23], blood vessels [24] and cartilage [25].

Cellulose is obtained from plants, algae, and tunicates—but mainly from wood [26]—by mechanical and chemical processes [27,28]. The extraction of cellulose from wood has been causing deforestation [29], and with the climate changes that the planet has been experiencing in recent years, it is of the utmost importance to reduce it. Currently, deforestation is occurring more in the Amazon, in Africa (Congo Basin) and in Indonesia, whose main causes are the massive production of soy and the exploitation of cellulose [30]. Thus, it is imperative to find wood-cellulose substitutes.

Studies developed by our group investigated potential substitutes for wood cellulose, such as cellulose extracted from agro-industrial residues. The study proved that it is possible to extract cellulose from corn husk, pomegranate peel, fava pod, grape stalk, strawberry-tree fruit marc and corncob. Among the sources studied, the highest percentage of cellulose was obtained from corncob, and its properties are quite similar to those of cellulose extracted from wood [31].

Natural polymers such as cellulose have several advantages as mentioned earlier, but unfortunately, due to rapid hydrolysis, they quickly lose their mechanical/structural properties. Alternatively, synthetic polymers offer good mechanical support and high reproducibility and processability. However, unlike natural polymers, they have the disadvantage that cells in contact with them do not maintain their phenotype and produce an extracellular matrix with inferior properties, limiting the regenerative potential of the scaffold [11]. In this sense, more recent studies have concluded that a hybrid scaffold, i.e., the combination of synthetic and natural materials, is the most viable option to guarantee all the desired properties [13]. Synthetic polymers commonly used in cartilage regeneration include poly(D, L-lactic-co-glycolic acid), poly(caprolactone) (PCL), poly(ethylene glycol) and poly(glycolic acid) [32]. PCL has excellent mechanical properties, is biodegradable, biocompatible, resorbable by the human body, FDA approved as implantable material, and has been extensively used for the repair of cartilage defects [33,34].

There are very few studies in the literature that describe the combination of PCL and cellulose through the extrusion technique. Alemán-Domínguez ME et al. (2018, 2019) studied the influence of cellulose concentration on scaffold properties and found that the incorporation of 2% of cellulose in PCL scaffolds results in higher mechanical properties (elastic modulus of flexure and compression) and better cell proliferation [35,36].

In this work, scaffolds composed of poly-Ɛ-caprolactone (PCL) and corncob cellulose (CC) were manufactured by extrusion using the fused-deposition-modeling technique, to replace wood cellulose with more sustainable cellulose. Thus, scaffolds with PCL and CC at two different levels of incorporation, 1% and 2% (*w/w*), were prepared and analyzed against control samples produced with commercially available wood cellulose (WC, microcrystalline cellulose). Thus, the manufactured composite scaffolds were characterized in terms of their morphological, chemical, and mechanical compressive properties. Additionally, their enzymatic degradation profile and in vitro biocompatibility were also assessed.

## 2. Materials and Methods

### 2.1. Scaffold Manufacturing

**Scheme 1:**
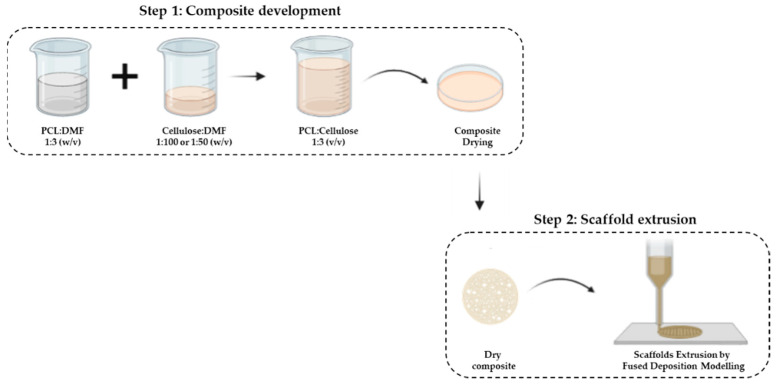
Composite development. Step 2: Scaffolds extrusion by fused-deposition-modeling (FDM) technique.

The manufacturing process of the composite scaffolds involves 2 steps (Scheme 1): (i) composite development (mixture of the 2 polymers) and (ii) extrusion of the scaffold.

Thus, the first step concerns the mixture of PCL with cellulose through the solvent-casting technique. First, PCL (Perstorp, Warrington, UK, MW = 50,000) was dispersed in dimethylformamide (DMF) (Chem-Lab, Zedelgem, Belgium) at a ratio of 1:3 (*w*/*v*) for 1 h at 80 °C under constant stirring. Then, corncob cellulose (CC), previously characterized by our group [31], was dispersed in DMF in the ratio of 1:100 and 1:50 (*w*/*v*) for 15 min at room temperature (RT) using an ultrasonic homogenizer (Hielsher UP200Ht). Finally, each cellulose dispersion was added to the PCL solution at a ratio of 1:3 (*v*/*v*) under constant stirring at 80 °C for 3 h. The mixture was deposited in Petri dishes and allowed to dry at RT for 7 days to evaporate the DMF. Commercial wood cellulose (WC) with PCL at the same concentrations were used as control samples.

The mixtures of PCL with cellulose were classified as follows: CC_1% and CC_2% represent the mixtures with 1% and 2% (*w*/*w*) corncob cellulose, respectively; WC_1% and WC_2% represent the mixtures with 1% and 2% (*w*/*w*) of wood cellulose, respectively.

In the second step, the extrusion of the scaffolds was performed by the fused-deposition-modeling (FDM) technique using the Bioextruder equipment previously developed by our group [37]. As the composite developed in step 1 was in the form of a membrane, it was cut into small pieces and melted in the extruder chamber.

The operational parameters used in the production of the scaffolds using the Bioextruder were 8 mm/s and 14.6 rpm spindle speed and material-flow speed, respectively. The temperatures of the melting chamber and the extrusion nozzle were set at 75 °C and 80 °C, respectively. The scaffold structure was designed in the form of a cylinder, with a diameter of 10 mm, composed of 10 layers with a fiber diameter of 300 µm and filament alignment of 0°/90°. The needle used to obtain this fiber diameter had a diameter of 22 ga/400 µm.

### 2.2. Differential-Scanning-Calorimetry (DSC) Analysis

To extrude the composites developed in step 1 of the scaffold manufacturing, it is necessary to consider their melting temperature, since the chamber where it will be placed must be at a temperature higher than the melting temperature of the material. Therefore, thermal analysis of the dried composites was performed using DSC on a Simultaneous Thermal Analyzer, STA 6000 system (Perkin Elmer, Waltham, MA, USA). The samples (approximately 6.5 mg) were weighed in alumina pans and heated from 20 °C to 150 °C at 10 °C/min, under a nitrogen-flow rate of 20 mL/min. Melting temperature was recorded and analyzed using PyrisTM software. This analysis was performed in triplicate.

### 2.3. Attenuated-Total-Reflectance–Fourier-Transform-Infrared (ATR–FTIR)-Spectroscopy Analysis

The dry composites obtained in step 1 of the fabrication of the scaffolds were analyzed by ATR–FTIR to confirm the complete evaporation of the solvent, DMF. A Bruker Alpha-P FTIR spectrometer was used in the absorbance mode, with ATR platinum–diamond coupling. The dry composites were analyzed at RT, with a spectral resolution of 4 cm^−1^ at 64 scans per sample, and in the range 4000–400 cm^−1^.

### 2.4. Morphological Analysis of the Scaffolds

The structure and surface morphology of the produced scaffolds were observed to obtain some important parameters for comparison with native cartilaginous tissue.

Thus, the scaffolds were morphologically analyzed by: (i) digital caliper (Wurth, Künzelsau, Germany) for diameter and thickness; (ii) optical microscopy (Micros, Gewerbezone, Austria) using the Microvisible software to obtain pore and filament dimensions; (iii) computerized microtomography (Micro-Computed Tomography, MicroCT, SkyScan 1174TM, Bruker, MA, USA) for porosity and interconnectivity assessment. Microscopic analysis was performed at 40× magnification. The MicroCT analysis was performed using a 50 kV/40 W X-ray source and a 1.3 megapixel X-ray camera. The parameters used for scanning the samples were: a step of 0.7 around the medio-lateral axis, resulting in 210 images; an acceleration voltage of 50 kV; a beam of 800 μA; an exposure time of 3500 ms; an image pixel size of 9.6 μm; without a filter.

### 2.5. Mechanical Analysis

The purpose of the scaffold is to replace damaged articular cartilage. Thus, it is necessary that the produced scaffolds exhibit mechanical properties similar to those of native tissue. The mechanical strength of the scaffolds was tested using a universal testing machine (Instron 5544, Instron, Norwood, MA, USA) at an extension rate of 1 mm/min. The compression modulus of elasticity (E) was calculated by the slope of the linear region in the stress–strain curve. Five samples of each scaffold composition were tested.

### 2.6. Enzymatic Degradation

Enzymes are naturally present in the human body. In the case of a traumatic situation in cartilage, lysozyme concentration is increased. Therefore, a degradation test with lysozyme illustrates the resistance of the produced scaffolds when in contact with this enzyme. The scaffolds of each formulation were incubated in phosphate-buffered-saline (PBS) medium (Alfa Aescar, Kandel, Germany), at pH 7.4, with a lysozyme (Sigma-Aldrich, St. Louis, MO, USA) concentration of 2 mg/mL [38]. The enzyme solution was prepared by adding the enzyme to the PBS medium under stirring at 100 rpm for 10 min. After preparation, the enzyme solution was included in each microplate well with the scaffold and incubated at 37 °C for 28 days. The enzymatic degradation of the scaffolds was evaluated by measuring its mechanical resistance to compression at days 1, 7, 14 and 28. Scaffolds with WC were used as controls and submitted to the same treatment. Mechanical tests throughout the assay were compared with the values obtained in the mechanical analysis of the untreated scaffold after production (called day 0). The assay was performed in triplicate.

### 2.7. In Vitro Cytotoxicity Test

The biocompatibility of the manufactured scaffolds was assessed using L929 mouse fibroblasts (ATCC number CCL-1) and following the ISO 10993-5 and ISO 10993-12 guidelines [39]. Prior to testing, manufactured PCL-based cellulose scaffolds were sterilized by overnight exposure to ultraviolet (UV) light, washing with ethanol 70%, and incubation with a 1% (*v/v*) antibiotic–antimycotic solution (Anti–Anti, Gibco™, Fisher Scientific, Waltham, MA, USA) in phosphate-buffered saline (PBS, Gibco™, Fisher Scientific, USA) for 3 h at RT (one wash every hour).

The scaffolds were evaluated by performing the indirect-extract test and direct-contact test. In both tests the negative control was prepared by culturing L929 fibroblasts on tissue-culture-polystyrene (TCPS) plates with Dulbecco’s Modified Eagle’s Medium (DMEM, Gibco™, Fisher Scientific, USA) supplemented with 10% (*v/v*) Fetal Bovine Serum (FBS, Life Technologies, Carlsbad, CA, USA) and with 1% Anti–Anti in an incubator at 37 °C/5% CO_2_. Latex was used as a positive control for cell death. L929 fibroblasts were seeded on TCPS plates at a cell density of 10^5^ cells/well and cultured for 24 h at 37 °C/5% CO_2_ to generate a confluent monolayer.

For the indirect test, the extracts were prepared by incubating the scaffolds in DMEM, 10% FBS and 1% Anti–Anti culture medium at a ratio of 0.2 g scaffold/mL for 72 h at 37 °C/5% CO_2_. This ratio ensures that the test sample covers one-tenth of the surface of the cell layer, according to ISO 10993-5:2009 [39]. After that, the culture medium was removed and L929 fibroblasts were exposed to the material extract’s conditioned medium for 72 h at 37 °C/5% CO_2_. Then, the extract-conditioned medium was removed and the 3-(4,5-dimethylthiazol-2-yl)-2-5 diphenyl tetrazolium bromide (MTT) assay (In Vitro Toxicology Assay Kit MTT based, Sigma-Aldrich, St. Louis, MO, USA) was performed in accordance with the manufacturer’s guidelines. Briefly, the cells were incubated with MTT solution (1 mg/mL, yellow) for 2 h at 37 °C, and afterwards the violet formazan product resulting from the metabolic reduction of MTT by the metabolically active cells was dissolved under stirring using a solution of 0.1 N HCl in anhydrous isopropanol (Sigma-Aldrich). The absorbance of the final solutions was measured in a plate reader (Infinite M200 PRO, TECAN, Mannedorf, Switzerland) at 570 nm.

In the case of the direct-contact assay, the scaffolds were carefully placed over the previously formed confluent monolayer of L929 fibroblasts (in the center of each of the replicate wells; three replicates were used for each sample) and incubated for 72 h at 37 °C/5% CO_2_, according to the ISO 10993-5:2009. Afterwards, cell viability and morphology were qualitatively evaluated under an inverted optical microscope (LEICA DMI3000B, Leica Microsystems, Wetzlar, Germany) equipped with a digital camera (Nikon DXM1200F, Nikon Instruments Inc., Melville, NY, USA) to assess any cytotoxic responses such as the occurrence of halo-inhibition effect or abnormal fibroblast morphology.

### 2.8. Statistical Analysis

Statistical analysis was performed using the GraphPad Prism 8 software (GraphPad Software, Inc., San Diego, CA, USA). One-way ANOVA with Tukey’s test was applied to analyze differences in morphological parameters (diameter and thickness), the mechanical properties and in vitro cytotoxicity results. A two-way ANOVA, with Tukey’s test, was applied to the mechanical tests performed after enzymatic degradation. In addition, the level of statistical significance was set as 95%, 99% and 99.9% (* *p* < 0.05, ** *p* < 0.01, *** *p* < 0.001).

## 3. Results

### 3.1. PCL–Cellulose Composites Characteristics

**Figure 1:**
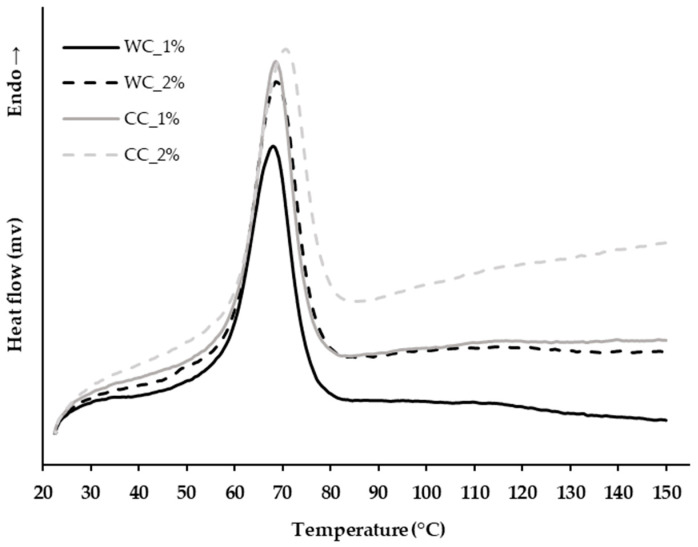
Thermal analysis by differential scanning calorimetry (DSC) of the PCL–cellulose composites produced in step 1 with: 1% wood cellulose (WC_1%); 2% wood cellulose (WC_2%); 1% corncob cellulose (CC_1%); 2% corncob cellulose (CC_2%).

The thermal properties of the PCL–cellulose composites developed in step 1 of the scaffold manufacturing (Section 2.1., Materials and Methods) were evaluated. As the PCL–cellulose composites must be melted in order to be extruded, their melting temperature was determined by controlled heating from 30 °C to 150 °C, and through this, the extruder chamber temperature was set. Figure 1 shows that all PCL–cellulose composites had similar melting temperatures, 65.31 ± 0.10 °C, 66.84 ± 1.2 °C, 66.53 ± 0.56 °C, and 68.64 ± 0.29 °C for WC_1%, WC_2%, CC_1% and CC_2%, respectively. The corncob cellulose increases the melting temperature in composites in relation to wood cellulose, and also by increasing its percentage in the formulation. Furthermore, it is possible to increase the extrusion temperature up to 150 °C and no degradation event will occur.

**Figure 2:**
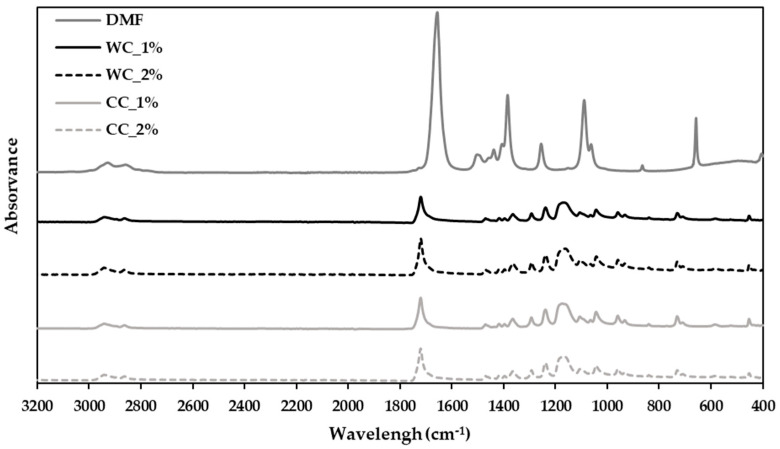
Attenuated-total-reflectance–Fourier-transform-infrared (ATR–FTIR)-spectroscopy analysis of the composites produced in step 1 with PCL and: 1% wood cellulose (WC_1%); 2% wood cellulose (WC_2%); 1% corncob cellulose (CC_1%); 2% corncob cellulose (CC_2%); and DMF as the solvent used in composite preparation.

For the PCL–cellulose-composite preparation, DMF was used as a solvent to solubilize and aggregate PCL and cellulose. A chemical analysis was performed using ATR–FTIR (Figure 2) to assess whether the DMF was completely removed and whether the composites could be extruded.

The FTIR spectrum (400 to 3200 cm^−1^) shows that the strongest band in DMF is the vC=O at 1673 cm^−1^ [40]. This band does not appear in any spectrum of the developed composites, demonstrating that DMF was fully evaporated from all composite samples, suggesting its readiness for extrusion.

### 3.2. Morphological Characteristics of the Scaffolds

Despite the configurations designed for the scaffolds, i.e., cylinders with 10 mm of diameter and 2.5 mm of height, the samples produced show some deviation, which is easily explained by the shrinkage/contraction of the material during solidification after extrusion (Table 1). The scaffolds’ diameter varied between 8.92–9.34 mm and their height ranged from 2.29–2.39 mm.

Thus, the scaffolds whose dimensions more closely approached the theoretical configuration were WC_2% and CC_1%, with 2.38 ± 0.10 mm and 2.39 ± 0.11 mm in height, respectively, and 9.27 ± 0.15 mm and 9.34 ± 0.15 mm in diameter, respectively.

**Figure 3:**
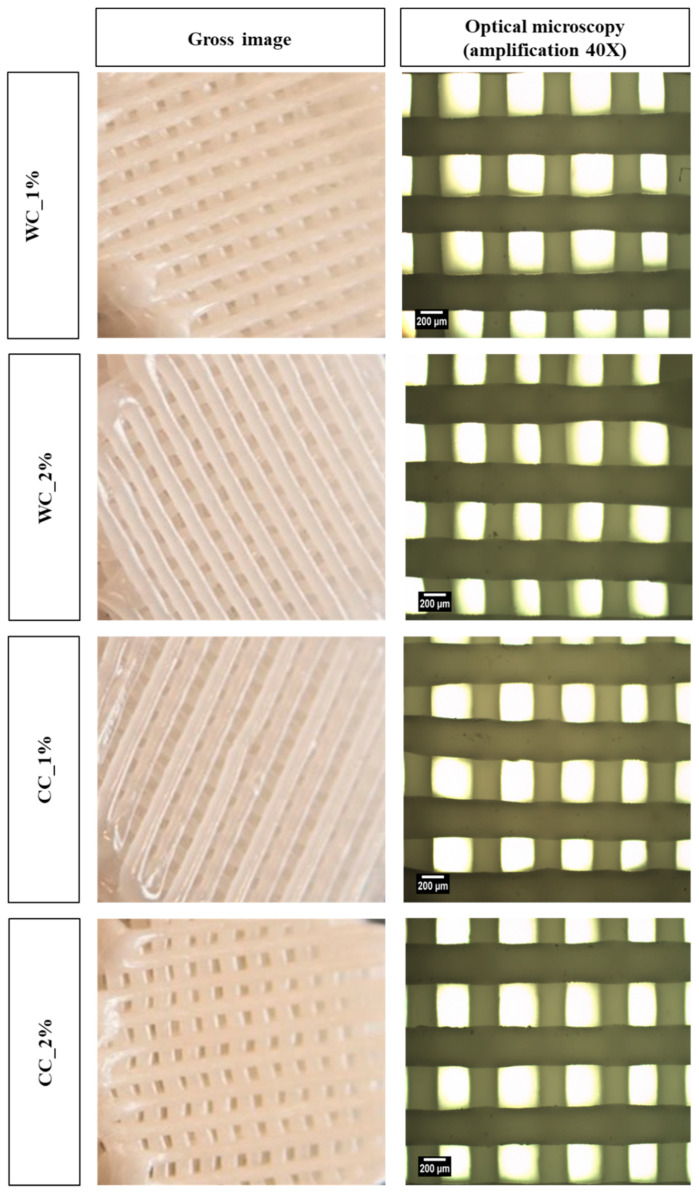
Morphology assessment: gross images (left column) and optical-microscope images (right column) (40× amplification) of scaffolds produced with PCL and: 1% wood cellulose (WC_1%); 2% wood cellulose (WC_2%); 1% corncob cellulose (CC_1%); 2% corncob cellulose (CC_2%).

**Figure 4:**
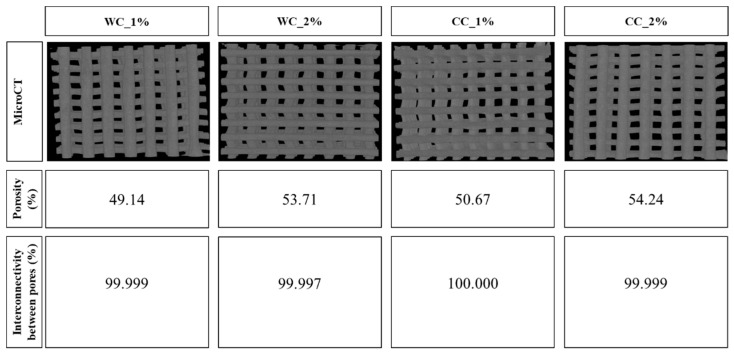
Porosity (%) and interconnectivity between pores (%) calculated through Skyscan MicroCT (CTAn software), of scaffolds produced with PCL and: 1% wood cellulose (WC_1%); 2% wood cellulose (WC_2%); 1% corncob cellulose (CC_1%); 2% corncob cellulose (CC_2%).

The surface of all scaffolds can be observed by naked eye and optical microscopy (Figure 3) and the internal and external 3D structure by MicroCT (Figure 4). Using optical microscopy at 40× magnification, it is possible to observe the upper layer of the scaffold, which provides more detailed information on the dimensions of the filaments and pores. Thus, it is possible to verify that both the pore and filament diameters were ~300 µm. On the other hand, the MicroCT images showed homogeneous samples, with no cellulose agglomerates being detected, either in WC or CC scaffolds.

The porosity of the tested scaffolds was very similar, ranging between 49% and 54%. The scaffold with the highest porosity was CC_2% (54.24%) as opposed to WC_1% (49.14%), which presented the lowest porosity. In addition, all samples showed interconnectivity percentages of approximately 100%, with the lowest being WC_2% (99.997%) and the largest being CC_1% (100.000%) (Figure 4).

### 3.3. Mechanical Properties of the Scaffolds

**Figure 5:**
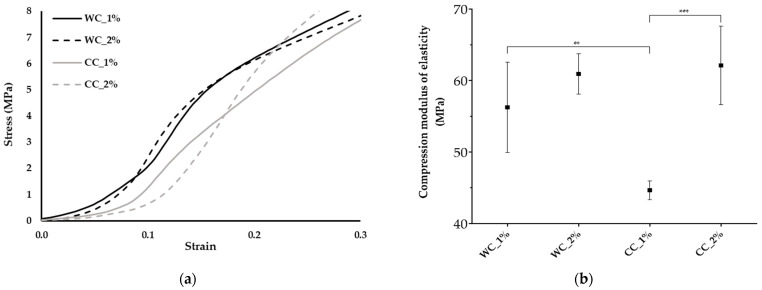
Mechanical properties: (**a**) Stress–strain curves and (**b**) compression modulus of elasticity of the scaffolds produced with PCL and: 1% wood cellulose (WC_1%); 2% wood cellulose (WC_2%); 1% corncob cellulose (CC_1%); 2% corncob cellulose (CC_2%). Measures of five samples were considered (*n* = 5). Statistically significant differences at *p* < 0.01 (**) and *p* < 0.001 (***).

The mechanical behavior of the scaffolds was evaluated under compressive testing (Figure 5), since the samples are intended for articular cartilage substitutes, in which the compressive loads are particularly relevant. Figure 5a shows the standard compression stress–strain curves for the different scaffolds, corresponding to the behavior of a thermoplastic material, with an initial elastic region followed by a plastic zone.

Through the analysis of the compression modulus of elasticity (Figure 5b), it appears that the use of WC, regardless of the degree of incorporation (1% or 2%), resulted in similar mechanical performance (56 ± 6.3 MPa and 61 ± 2.8 MPa, respectively). However, in CC scaffolds, the use of 2% significantly increased the compression modulus of elasticity (44 ± 1.3 MPa for CC_1% and 62 ± 5.5 MPa for CC_2%). It was possible to observe significantly lower mechanical properties during compression in the case of CC_1%, while CC_2% presented similar values to WC. In addition, the yield stress (Figure 5a) was the highest for the CC_2%, demonstrating a better response to higher loads.

### 3.4. Enzymatic Degradation of the Scaffolds

When traumatic situations occur, lysozyme levels in articular cartilage increase [41]. Therefore, an enzymatic-degradation assay was performed and the mechanical properties of the scaffolds under compression were monitored on days 1, 7, 14 and 28 of the experiment and compared to day 0 (Table 2).

During enzymatic degradation, the only sample that showed changes in its mechanical properties was WC_1% with a decrease in its compression modulus of elasticity from day 0. Throughout the test, WC_1% presented the lowest modulus in relation to the other scaffolds (WC_2% and CC_1%). CC_1% and CC_2% presented statistically similar performances.

**Figure 6:**
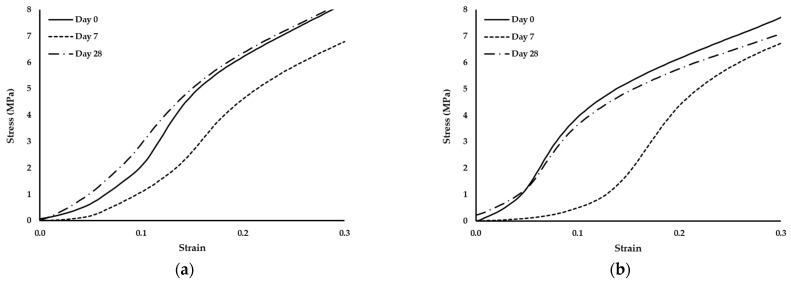

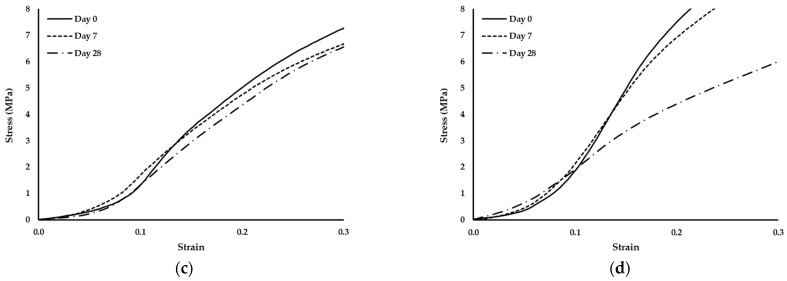
Stress–strain curves of scaffolds produced with PCL and: (**a**) 1% wood cellulose (WC_1%); (**b**) 2% wood cellulose (WC_2%); (**c**) 1% corncob cellulose (CC_1%); (**d**) 2% corncob cellulose (CC_2%) on days 0, 7 and 28 of enzymatic degradation with lysozyme.

Since no degradation effects were detected through the analysis of the compressive modulus of elasticity, the standard compression curves of the scaffolds were evaluated to validate this fact. Thus, Figure 6 depicted the curves of the scaffolds on days 0, 7 and 28.

It should also be noted that the scaffold manufactured with WC exhibited the same behavior regarding the yield stress. At day 7 they presented the highest yield stress but at day 28 it decreased to values similar to day 0. However, when scaffolds were manufactured with CC, the behavior changed, with a decrease in the yield stress over time.

### 3.5. In Vitro Cytocompatibility of the Scaffolds

**Figure 7:**
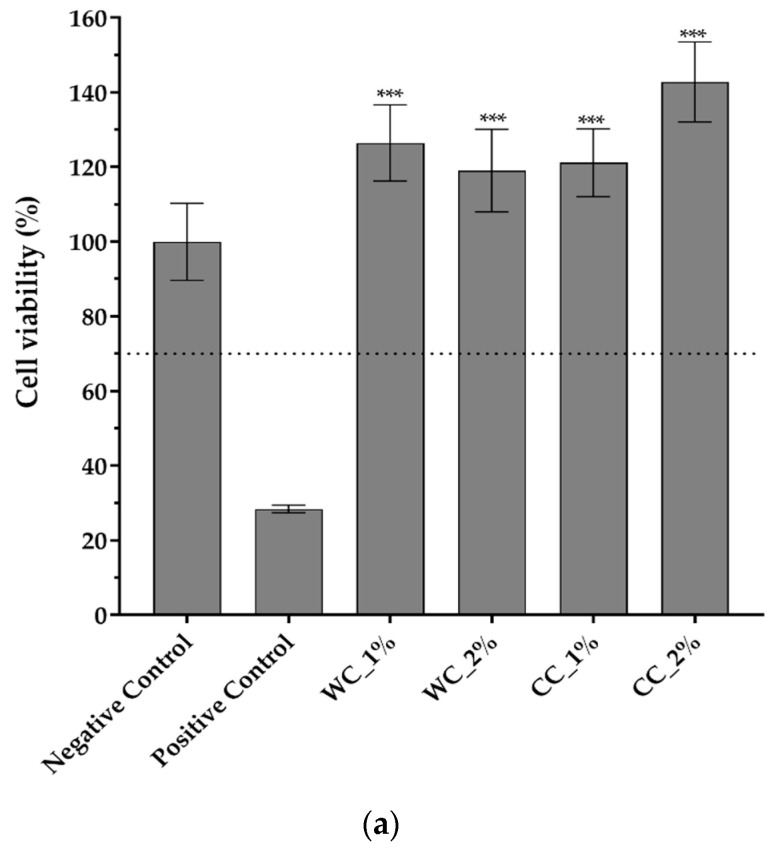

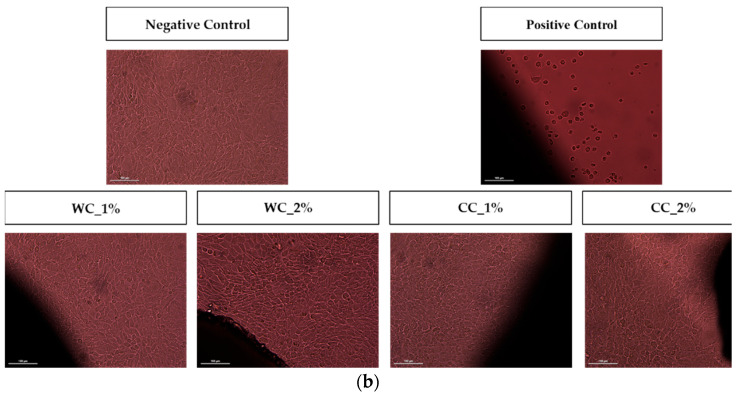
Cytotoxicity assay with L929 mouse fibroblasts through (**a**) indirect contact (MTT protocol) (*n* = 5) and (**b**) direct contact (20× amplification) of negative and positive controls and the scaffolds produced with PCL and: 1% wood cellulose (WC_1%); 2% wood cellulose (WC_2%); 1% corncob cellulose (CC_1%); 2% corncob cellulose (CC_2%). Scale bar: 100 μm. *p* < 0.001 (***).

Since the purpose of scaffolds is to support damaged cartilaginous tissue to aid in its recovery, it is necessary to evaluate them in terms of their cytotoxicity effect. Figure 7 shows the cytotoxicity results of indirect- and direct-contact tests performed with L929 fibroblast cells. Figure 7a shows that not only are all PCL–cellulose scaffolds not cytotoxic but, additionally, that they promote cell viability/proliferation, since all viability values were higher than the negative control (>100%), with the highest value corresponding to the CC_2% scaffold (142.8 ± 10.7%). The direct-contact test (Figure 7b) demonstrated that, after 72 h of contact between the scaffolds and fibroblast-confluent cultures, WC_1%, WC_2%, CC_1% and CC_2% presented viable cells with a typical spindle-shaped morphology, in which no halo-inhibiting effect was observed.

## 4. Discussion

Previous work by our group [31] proved that corncob, an agro-industrial byproduct, can be a viable option as a source of cellulose to replace wood cellulose due to its characteristics. To evaluate the performance of the corncob cellulose in real applications against commercial wood cellulose, PCL-based scaffolds were produced with different percentages of cellulose using the additive manufacturing technique (extrusion).

Before scaffold extrusion, it is necessary to access the properties of the developed composite materials. Thermal characterization by DSC was performed to determine the melting temperature of the composites and predict the occurrence of any undesired event in the extrusion temperature range. Figure 1 demonstrates that the melting temperature of all developed PCL–cellulose composites was around 65 °C, which is approximately the melting temperature of PCL [36].

Regarding the use of DMF as a solvent to combine PCL with cellulose during the development of the composite, its complete evaporation is essential to in order not to affect the extrusion process and not to be retained in the polymeric network of the scaffolds, which causes cytotoxicity, since DMF is a very toxic organic solvent for cells [42]. The ATR–FTIR analysis (Figure 2) demonstrated that DMF was fully removed from the composites since the band at 1673 cm^−1^, which concerns the link vC=O, does not appear in the composites’ spectra. Thus, it is possible to safely extrude the scaffolds and they will probably not exhibit cytotoxicity.

The scaffolds were designed with a cylindrical shape, facilitating their incorporation into the knee as a cartilage substitute. Some deviations from the initial designed CAD configurations were observed. These were due to the temperature variations to which the composites were submitted during melting, causing a certain material shrinkage from the viscous to the solid state, which justifies the smaller dimensions observed. There were also differences between the height of the scaffolds, which are also justified by the fact that, ideally, the layers are tangential to each other, with a cylindrical filament geometry of 300 µm diameter. However, in practice and to ensure adhesion between the layers, the increment in the Z axis is not 300 µm, but 280 µm (approximately 7% less than the total filament height). Accordingly, the shrinkage of the material causes a slight reduction in the height of the scaffold. In this study, in terms of dimensions, the CC_1% and WC_2% scaffolds presented less statistically significant differences when compared to the CAD-design models.

Scaffolds at 40× magnification showed a detailed top layer that allowed the determination of filament and pore dimensions. Thus, a pore and filament diameter of ~300 µm was obtained, which is in the range of the desired dimensions for cell adhesion and proliferation. The literature results suggest that chondrocytes present in the articular cartilage [43] show a preference for matrices with pore sizes ranging from 200 µm to 405 µm [44,45]. Additionally, studies that developed different pore sizes in the same scaffold indicated that the cell adhesion was greater in the center of the scaffolds, where the pore size was 390 µm [46]. Furthermore, all the surfaces of the scaffolds were shown to have a smooth surface, as indicated in the study by Alemán-Domínguez ME et al. (2019), for samples with cellulose concentrations below 5% [36].

It is important to verify that the cellulose is well dispersed throughout the scaffold, avoiding cellulose agglomerates. MicroCT analysis shows that no cellulose agglomerates were detected, suggesting that the cellulose from wood or corncob was well dispersed within the scaffolds. The porosity results of each scaffold determined through MicroCT showed a maximum variation of 5% (from 49 and 54%). Despite this, the porosity of the scaffold was positively correlated with the increase in the cellulose content, as indicated in other studies [35]. CC_2% stands out for its higher porosity; however, it was not close to the desired value for chondrocyte proliferation, which is between 77% and 92% [47]. However, a previous study observed a porosity for PCL scaffolds of 57%, which is close to that observed in the CC_2% scaffold. This value was estimated considering the biodegradation of the material and its mechanical and biological requirements [48]. All samples presented interconnectivity percentages of approximately 100%, indicating that cells can migrate and proliferate throughout the entire scaffold [49]. Interestingly, despite the low porosity, the pore size and interconnectivity values were within the ideal range for chondrocyte adhesion/proliferation [50].

As the purpose of scaffolds, such as those developed in this work, is the replacement/repair of hyaline cartilage (e.g., knee), understanding its response to compressive loads is extremely important. According to Shepherd (1999), the compression modulus of elasticity for human cartilage in the knee is between 5.5 and 11.8 MPa [51]. The results revealed no improvement of the compression modulus of elasticity for the incorporation of 1% or 2% of WC (56 ± 6.3 and 61 ± 2.8 MPa, respectively). Comparing the two types of cellulose at 1%, CC scaffolds showed a significant decrease of about 21% (45 ± 1.3 MPa, *p* < 0.01) over WC scaffolds. This occurred due to the fact that WC is morphologically more homogeneous in terms of particle size and has a more regular/smoother surface than CC [31]. These particle differences result in distinct mechanical properties between the celluloses, where heterogeneous particles create greater instability in the structure, thereby reducing its mechanical properties. Furthermore, after increasing the cellulose content from 1% to 2%, there was a significant increase of about 40% (*p* < 0.05). Regarding the results obtained from the two different sources of cellulose at 2%, similar values were found, 61 ± 2.8 MPa for WC and 62 ± 5.5 MPa for CC, suggesting that corncob cellulose is a viable alternative to cellulose from wood, reducing agro-industrial waste and contributing to a more sustainable approach for a biotechnological application.

In general, CC (2%) endows PCL-based scaffolds with mechanical properties equal to WC, thus functioning as a good substitute. However, in addition to ensuring the mechanical performance of the scaffolds at the time of their implantation, evaluating their performance over time under in vivo conditions is also very important. Since the final biomedical application of the scaffold is to replace damaged cartilage, which has a higher concentration of lysozyme when injured [41], it is important to assess the performance of the scaffold in contact with this enzyme.

During the 28 days of enzymatic degradation, all scaffolds presented statistically similar values of the compression modulus of elasticity, with the exception of WC_1%, which reduced its modulus on the first day of testing. This translates into a mechanical stability of the scaffold structure even after exposure to lysozyme, except for WC_1%. Observing the values of the compression modulus of elasticity, WC_2% presented higher values at each day of testing, indicating that the concentration of cellulose influenced the mechanical resistance of the scaffold throughout the entire experiment. However, with regard to CC, the increase in its concentration did not influence its mechanical strength when subjected to enzymatic degradation, with CC_1% and CC_2% being statistically equal over time. This result proves that corncob cellulose can be used in smaller amounts in the manufacturing of scaffolds and the mechanical response will be similar. Nonetheless, CC_1% presented mechanical values of compression that were higher than WC_1%, (*p* < 0.05) but that were statistically different only on day 7. However, CC_2% was the most similar in mechanical terms to WC_2%, but with a lower compression modulus of elasticity on day 7. Thus, scaffolds with CC_2% contain an ideal percentage for application as a viable replacement for WC. Since lysozyme is an antibacterial enzyme capable of degrading the β-1,4-linkages between N-acetylmuramic acid and N-acetyl-d-glucosamine, which are found in the bacterial cell wall [52], no degradation by the action of lysozyme in the scaffolds is expected. In fact, no reduction or change in the structure of the developed scaffolds was noticed, since the two polymers applied in this study, PCL and cellulose, do not contain that specific chemical bond. This was further validated by the analysis of the standard stress–strain curves of the scaffolds exposed to lysozyme at days 0, 7 and 28. Although the yield stress decreased, the thermoplastic behavior persisted, with the observation of an elastic region followed by a plastic one.

Regarding the cytotoxicity tests, the results suggested the in vitro cytocompatibility of the developed scaffolds. Previous studies performed with PCL and commercial cellulose scaffolds at different concentrations (2, 5 and 10%) indicated that cellulose does not affect cell adhesion; however, cell proliferation increases when cellulose concentration is 2% [36]. These results corroborate those obtained in this study. All the scaffolds, with both commercial and corncob cellulose, did not exhibit cytotoxicity and, additionally, potentiated the viability/proliferation of the L929 fibroblasts used in the analysis. These results also reinforce those obtained through FTIR analysis, proving that all DMF was removed during the drying process of the composites.

Regarding WC, increasing the concentration decreases cell viability, as previously described by Alemán-Domínguez, M.E., (2019) [36]. However, when CC is used, the increase in concentration increases cell viability to levels higher than WC. Fibroblast morphology was also not altered, and no halo of inhibition was detected, indicating that all the produced scaffolds have high potential for cartilage-regeneration applications both in vitro and in vivo. Future work will include the evaluation of the biological performance of PCL–CC-composite scaffolds in the context of cartilage tissue engineering by culturing them with human chondrocytes as well as assessing their ability to promote the chondrogenic differentiation of human mesenchymal stem/stromal cells.

## 5. Conclusions

The development of PCL–cellulose scaffolds to repair damaged cartilage was studied with corncob cellulose as an alternative to wood cellulose. Scaffolds with corncob cellulose presented morphological and mechanical properties similar or better to scaffolds with wood cellulose. The concentration of 2% of corncob cellulose presented the best results, proving to be the optimal concentration. The enzymatic degradation revealed that CC_2% presents a compression modulus of elasticity very similar to WC_2%, with both scaffolds resisting degradation over time. Regarding the cytotoxicity assay, it was revealed that not only are all manufactured scaffolds not cytotoxic, but they also promote cell viability/proliferation. CC_2% presents the highest cell viability, suggesting its advantageous biological performance over WC. Overall, regarding the properties assessed in this study, the scaffolds developed with 2% corncob cellulose seem to have high potential to be applied as novel biocompatible and renewable materials for articular-cartilage-replacement strategies.

## Figures and Tables

**Table 1:** Dimensions of the scaffolds produced with PCL and: 1% of WC (WC_1%); 2% of WC (WC_2%); 1% of CC (CC_1%); 2% of CC (CC_2%). (*n* = 9).

Scaffolds	Height (mm)	Diameter (mm)
CAD Design	2.50	10.00
WC_1%	2.34 ± 0.09 ***	8.92 ± 0.12 ***
WC_2%	2.38 ± 0.10 *	9.27 ± 0.15
CC_1%	2.39 ± 0.11 *	9.34 ± 0.15
CC_2%	2.29 ± 0.05 ***	9.12 ± 0.13 ***

Statistically significant differences at *p* < 0.05 (*) and *p* < 0.001 (***).

**Table 2:** Compression modulus of elasticity (MPa) of scaffolds produced with PCL and: 1% wood cellulose (WC_1%); 2% wood cellulose (WC_2%); 1% corncob cellulose (CC_1%); 2% corncob cellulose (CC_2%) during enzymatic degradation with lysozyme. (*n* = 3).

Time (Days)	Compressive Modulus (MPa)			
	WC_1%	WC_2%	CC_1%	CC_2%
0	55.55 ± 4.67 ^A,a^	60.76 ± 2.11	44.54 ± 0.82 ^b,c^	63.31 ± 3.95 ^d^
1	47.66 ± 4.63 ^B,a^	58.18 ± 3.12 ^b^	47.74 ± 8.02	53.88 ± 3.76
7	35.32 ± 3.26 ^a^	56.00 ± 2.99 ^b,c^	47.52 ± 2.29 ^b^	42.95 ± 1.35 ^d^
14	37.19 ± 1.27 ^a^	59.48 ± 0.25 ^b^	51.91 ± 7.32 ^b^	48.47 ± 9.84
28	43.77 ± 4.17 ^a^	59.01 ± 3.48 ^b^	48.50 ± 7.89	54.40 ± 8.91

^A–B^ Different capital letters mean significant differences (*p* < 0.001) over time. ^a–b, c–d^ Different small letters mean significant differences (*p* < 0.05) among scaffolds each time.

## Data Availability

Not applicable.
